# Anti-atherosclerotic effect of traditional fermented cheese whey in atherosclerotic rabbits and identification of probiotics

**DOI:** 10.1186/s12906-016-1285-8

**Published:** 2016-08-24

**Authors:** Xin-Hua Nabi, Chun-Yan Ma, Tabusi Manaer, Mulalibieke Heizati, Baheti Wulazibieke, Latipa Aierken

**Affiliations:** Department of Pharmacology, Xinjiang Medical University, Urumqi, 830011 China

**Keywords:** Traditional fermented cheese whey, Atherosclerosis, Vascular cellular adhesion molecule-1, Intercellular cellular adhesion molecule-1, C-reactive protein, Lipid profile, Lactic acid bacteria, Yeast

## Abstract

**Background:**

Traditional fermented cheese whey (TFCW), containing probiotics, has been used both as a dairy food with ethnic flavor and a medicine for cardiovascular disease, especially regulating blood lipid among Kazakh. We therefore investigated anti-atherosclerotic effects of TFCW in atherosclerotic rabbits and identified lactic acid bacteria (LAB) and yeasts in TFCW.

**Methods:**

Atherosclerotic rabbits were induced by administration of atherosclerotic diet for 12 weeks and divided randomly into three groups and treated for 4 weeks with Simvastatin (20 mg/kg) or TFCW (25 mg/kg) and (50 mg/kg). In addition, a normal control group and an atherosclerotic group were used for comparison. All drugs were intragastrical administered once daily 10 mL/kg for 4 weeks. Body weight (BW), lipid profiles, C-reactive protein (CRP), vascular cell adhesion molecule-1 (VCAM-1), and intercellular adhesion molecule-1 (ICAM-1) were tested and theromatous plaques and the number of foam cells and infiltrating fibroblast cells in the thoracic aorta endothelium was evaluated by hematoxylin and eosin stainin. LAB and yeasts were isolated and purified by conventional techniques and identified using morphological and biochemical properties as well as gene sequences analysis.

**Results:**

After 4 weeks of treatment, high and low dose TFCW decreased serum TC, TG, LDLC, CRP, VCAM-1 and ICAM-1 (*P* < 0.05) compared to atherosclerotic group, and increased HDL-C (*P* < 0.05) compared to normal controls. Histological analysis showed TFCW reduced VCAM-1 expression and formation of atheromatous plaques on the aortic endothelium of atherosclerotic rabbits.

**Conclusion:**

Seven classes of LBA from two different genera including Lactobacillus brevis, Lactobacillus kefianofaciens, Lactobacillus helveticus, Lactobacillus Casei, Lactobacillus plantarum, Lactobacillus kefiri and Lactococcus lactic as well as 2 classes of yeasts from two different genera including Saccharomyces unisporus and Issatchenkia orientalis were isolated and identified from TFCW. In summary, TFCW, containing 7 classes of LBA and 2 classes of yeasts, has significant anti-atherosclerotic potential in atherosclerotic rabbits and may modulate lipid metabolism and protect aorta in the atherosclerotic condition, which might be related to various probiotics acting through reducing the CRP, VCAM-1 and ICAM-1 levels and protecting the aortic endothelium.

## Background

The public health burden of cardiovascular disease (CVD) is substantial, as CVD remains the leading cause of mortality and morbidity worldwide and atherosclerosis is the major cause of CVD [1, 2].

Traditional fermented cheese whey (TFCW), by-product of cheese-making, has been widely used as a traditional dairy medication for regulating blood lipid among Kazakh people [3]. Indeed, active peptides in TFCW up-regulate expression of peroxisome proliferator-activated receptor-γ (PPAR-γ) mRNA [3], which reduces atherosclerosis [4]. Furthermore, whey protein and peptides have a protective effect against CVD risk factors [5].

Probiotics mainly include *Lactobacillus* and *Bifidobacterium* and a few yeast species including *Saccharomyces boulardi* [6]. Lactic acid bacteria (LAB) are the main probiotics that prevent formation of aortic fatty lesions by inhibiting low-density lipoprotein (LDL) oxidation [7] and atherosclerosis via the inhibition of intestinal cholesterol absorption [8] in animal models. *S. boulardii*, one of the probiotic yeasts, provides anti-inflammatory and host immunity stimulatory effects [9] and lowers remnant lipoprotein, a highly atherogenic lipoprotein particle, in human adults with hypercholesterolemia [10].

However, anti-atherosclerotic effects of TFCW have not been experimentally demonstrated and no LAB or yeast has been found in TFCW. The aims of this study were to investigate anti-atherosclerotic effects of TFCW in a rabbit model of atherosclerosis and to identify LBA and yeast in TFCW.

## Methods

### Traditional fermented cheese whey (TFCW) manufacturing

Traditional fermented cow’s milk is the source of the cheese whey. Experimental TFCW samples were manufactured by standard procedures in 10 L vats in Altay Kanas Dairy Co. Ltd., (Altay, Xinjiang, China). Fresh cow’s milk samples were obtained from Jimunai Saur farm (Altay, Xinjiang, China) and skimmed in centrifuging at 3000 × g for 30 min, homogenized under the pressure of 1.5 ~ 1.7 Mpa and pasteurized by high temperature short time (HTST) then cooled to about 30 °C and fermented by inoculation with traditional home made Kazak yogurt purchased from Jimunai Saur farm (Altay, Xinjiang, China) at 37 °C for 12 h. After ferment, the whey was filtered in sterile gauze and dialyzed in cellulose membrane (12 kDa, Sigma) under constant magnetic stirring at 8 °C, also performed lactose removal by periodic water exchange. The experimental TFCW was stored at −20 °C until further use.

### Chemicals and reagents

Sodium pentobarbital was purchased from Merck & Co., (Germany). Simvastatin was purchased from Merck Sharp & Dohme (Australia) Pty Ltd., (Hangzhou, China). VCAM-1, ICAM-1 and CRP ELISA kits were purchased from Shanghai Senxiong Technology Co. Ltd., (Shanghai, China). Man Rogosa Sharpe (MRS) was purchased from Merck Sharp & Dohme (Australia) Pty Ltd., (Hangzhou, China). All media for cultivation of zymocytes were purchased from Hangzhou Microbial Reagent Co. Ltd., (Hangzhou, China).

### Animals and treatment

Sixty male white New Zealand rabbits, weighing 1.95-2.05 kg, specific pathogen free (SPF), were provided by Experimental Animal Center of Xinjiang Medical University, China and placed in separate cages and maintained on a 12-h day/night cycle at an ambient temperature, with *ad libitum* access to food and water. After a week of adaptive feeding, all the rabbits were randomly divided into 5 groups with 12 in normal group and 12 in atherogenic group, the normal control groups were given regular die and the atherogenic models were developed using an atherogenic diet for 12 weeks. The atherogenic diet consisted of 3 % cholesterol, 0.5 % sodium taurocholate, 0.2 % propylthiouracil, 5 % sugar, 10 % lard, and 81.3 % standard laboratory rabbit chow, which were provided by Experimental Animal Center of Xinjiang Medical University, China. After developing atherogenic models, Group 1 (normal control) was treated with saline in a matched volume; Group 2 (atherogenic group) had atherogenic rabbits treated with saline in a matched volume; Group 3 (positive control) had atherogenic rabbits administered with simvastatin 20 mg/kg; Group 4 and Group 5 were treated with TFCW 25 mg/kg and 50 mg/kg, respectively (low and high doses). Simvastatin and TFCW were intragastrical administered once daily 10 mL/kg for 4 weeks. All animals received care in compliance with the Chinese Convention on Animal Care, and the study was approved by the Institutional Ethics Committee of Xinjiang Medical University.

### Collection of blood and biochemical measurement

At the end of experiments, all rabbits were fasted for 12 h, weighed, anesthetized with sodium pentobarbital (Merck & Co.,) and continually monitored until total loss of consciousness as indicated by a total lack of response after a foot pinch. Blood samples were collected from abdominal aorta, allowed to clot on ice and subsequently subjected to centrifugation (3500 rpm at 4 °C for 10 min), where after serum aliquots were stored at −80 °C for further analysis. Serum TC, TG, LDL-C and HDL-C were examined via an automatic biochemical analyzer (BS-120, Shenzhen Mindray High-Tech Co., Ltd. China). CRP was determined by rate nephelometry (Beckman Coulter, USA). Serum ICAM-1 and VCAM-1 were determined using commercially-available ELISA kits according to manufacturer instruction.

### Histopathological study of aorta

Aorta was harvested from rabbits, placed immediately in formaldehyde 10 %, embedded in paraffin 24 h later, cut at 5 μm, stained with hematoxylin and eosin (H&E), and then scanned to assess pathological changes. For immunohistochemical staining, sections were incubated with anti-VCAM-1 (R&D Systems, MN, USA) and anti-F4/80 (Abcam, MA, USA) at 37 °C for 1 h, color developed with 3,3′-diaminobenzidine tetrahydrochloride and counterstained with hematoxylin. Samples in the absence of the primary antibodies were used as negative controls. Slides were observed under a light microscope, and images were subjected to statistical evaluation of positively stained cells in 10 random fields of view at a magnification of × 400. The average numbers of positively stained cells were counted per high power field (HPF).

### Isolation, purification and characterization of LAB

Agar plates with Man Rogosa Sharpe (MRS) broth suitable for lactobacillus growth were used for initial isolation of LAB single colonies. Single bacterial colonies were initially separated based on their morphological differences on agar plates. Cell morphology was observed under light microscopy after Gram staining. Catalase activity, carbohydrate fermentation, acidogenicity, aciduricity (final pH), and gas (CO_2_) production were analyzed. All isolates were presumptively identified as LAB strains based on their ability to grow on MRS agar plates, Gram-positive staining, and a catalase activity-negative phenotype [11]. 16S rDNA and 16S rRNA of 7 isolates were initially analyzed by BLAST program on NCBI website to search for the best matches among existing data in GenBank. 16S rDNA and 16S rRNA gene sequence analyses were carried out at Institute of Microbiology, Chinese Academy of Sciences, Beijing, China.

### Isolation, purification and identification of yeasts

Each 100 μl sample was enriched in a tube containing Sabourauds agar medium, incubated at 25 °C for 48-72 h and spread on Sabourauds agar. Representative yeast colonies were selected based on colonial characteristics, purified using a single colony isolation method, and maintained on a Sabourauds agar slant at 4 °C or in freezing tubes containing Sabourauds agar broth supplemented with 10 % glycerol at −80 °C. Physiological and biochemical characteristic identifications were made according to results of carbohydrate fermentation, carbon source assimilation, nitrogen assimilation and temperature tests. All these tests and analyses of 26S rDNA D1/D2 gene sequences were performed using identical methods as those used for bacteria.

### Statistical analysis

All values were reported as mean ± S.E.M. Data were analyzed by one-way ANOVA using SPSS 18 (SPSS Inc., Chicago, Illinois, USA). Significance was defined as ^*^P < 0.05 compared to atherogenic group.

## Results

### Effects of TFCW on Lipid Profiles

Table 1 showed serum lipid profiles among different experimental groups. Serum TC, TG, HDL-C and LDL-C of rabbits were significantly higher in atherogenic group than in normal control group (*p* < 0.05), whereas Simvastatin group and low and high dose TFCW group showed significantly lower serum TC, TG and LDL-C than did atherogenic group (*p* < 0.05). TFCW significantly increased HDL-C levels compared to normal control group (*p* < 0.05), but no significant difference was observed in treated groups with TFCW, compared with atherogenic group. Data indicated that TFCW affects lipid metabolic parameters and TFCW treatment could effectively improve lipid metabolism in atherogenic rabbits.

**Table 1:** Effect of TFCW on TC, TG, LDL-C and HDL-C in atherosclerotic rabbits (mmol/L, *n* = 12)

Group	TC	TG	HDL-C	LDL-C
Normal control	4.75 ± 2.00	0.53 ± 0.16	1.03 ± 0.22	0.18 ± 0.03
Atherogenic group	22.79 ± 2.13^*^	3.20 ± 1.32^*^	2.20 ± 0.95^*^	15.85 ± 3.2^*^
Simvastatin (20 mg/kg)	17.55 ± 1.6**	0.86 ± 0.22**	2.55 ± 0.23^*^	7.23 ± 2.87**
TFCW (25 mg/kg)	16.74 ± 3.4**	1.49 ± 0.73**	2.16 ± 0.69^*^	5.52 ± 1.76**
TFCW (50 mg/kg)	15.20 ± 5.25**	0.50 ± 0.20**	2.33 ± 0.18^*^	6.7 ± 2.28**

^*^ *P* < 0.05 vs. Normal control

**P <0.05 vs. Atherogenic group

### Effects of TFCW on CRP levels and body weight

Serum CRP was measured to determine inflammatory status of experimental groups as shown in Table 2. Generally, atherogenic group had significantly higher CRP, compared with the normal control group (15.03 ± 9.12 vs 0.98 ± 0.03 mg/L, *P* < 0.05). Outstandingly, the simvastatin and low and high dose TFCW groups had significantly lower CRP (*P* < 0.05) at values 3.43 ± 0.80 mg/L, 3.33 ± 0.50 mg/L and 1.34 ± 0.90 mg/L, respectively, compared with the atherogenic group. CRP in low and high dose TFCW was significantly decreased, compared to atherogenic group (*P* < 0.05). Data indicate that TFCW affects CRP and TFCW treatment could effectively improve inflammatory status in atherogenic rabbits. No significant differences were recorded in treated groups with TFCW on body weight compared with atherogenic group.

**Table 2:** Effect of TFCW on ICAM-1, VCAM-1, CRP and bodyweight in atherosclerotic rabbits (*n* = 12)

Group	CRP (mg/L)	Body weight (kg)	ICAM-1 (μg/L)	VCAM-1 (μg/L)
Normal control	0.98 ± 0.03	3.21 ± 0.37	32.89 ± 16.00	16.90 ± 6.03
Atherogenic group	15.03 ± 9.12^*^	3.03 ± 0.63	118.50 ± 30.12^*^	51.23 ± 9.00^*^
Simvastatin (20 mg/kg)	3.43 ± 0.80**	2.90 ± 0.15	63.65 ± 28.98**	41.14 ± 7.81**
TFCW (25 mg/kg)	3.33 ± 0.50**	2.35 ± 0.30	75.60 ± 28.40**	41.12 ± 8.90**
TFCW (50 mg/kg)	1.34 ± 0.90**	3.03 ± 0.30	42.25 ± 17.80**	41.75 ± 12.32**

^*^ *P* < 0.05 vs. Normal control

**P < 0.05 vs. Atherogenic group

### Effects of TFCW on ICAM-1 and VCAM-1

Adhesion molecules ICAM-1 and VCAM-1 play important roles in perpetuation of inflammation. Table 2 showed serum ICAM-1 and VCAM-1 among different experimental groups. ICAM-1 and VCAM-1 of rabbits were significantly higher in atherogenic group than in normal control group (*p* < 0.05), while Simvastatin and low and high dose TFCW group showed significantly decreased ICAM-1 and VCAM-1, compared to atherogenic group (*p* < 0.05). Data indicated that TFCW might affect ICAM-1 and VCAM-1 and TFCW treatment might effectively inhibit adhesion of circulating inflammatory cells to endothelial cell walls in atherogenic rabbits.

### Effects of TFCW on histopathological changes in the aorta

**Fig. 1:**
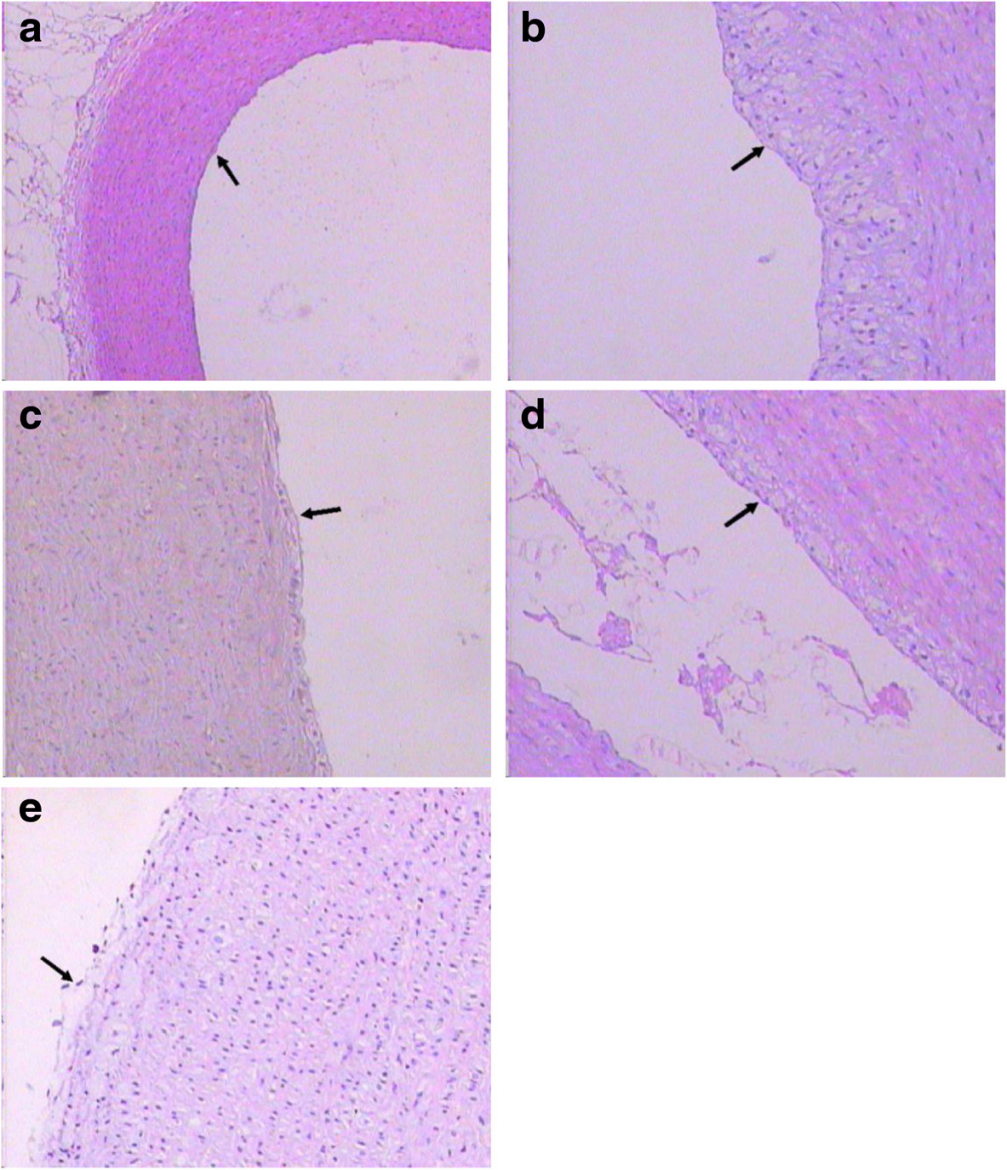
The effect of TFCW on histological sections of aorta in atherosclerotic rabbits (H&E). **a** Normal control; (**b**) atherosclerotic group; (**c**) positive control (simvastatin 20 mg/kg); (**d**) low dose TFCW (25 mg/kg); (**e**) high dose TFCW (50 mg/kg). (Magnification 400×)

Histopathological examination of aorta sections from experimental groups were shown in Fig. 1. Histological examination of hematoxylin and eosin-stained section of aorta of normal control showed normal histological examination of aorta, with no aortic lesion or presence of foam cells, while the aorta examination of atherogenic group showed thickening of intimal layer and accumulation of lipids, leading to formation of foam cells, signaling the early phase in development of atherosclerosis. Aorta of groups treated with simvastatin and low and high dose TFCW group showed reduced accumulation of atherosclerotic lesion due to decreased number of foam cells and cholesterol deposits in the aorta. Morphometric analysis of aorta section had showed that thickening of intimal layer and foam cells was increased in atherogenic group, compared to normal control group. Furthermore, groups treated with simvastatin and low and high dose TFCW showed decreased intimal layer and foam cells, compared with atherogenic group.

### Effects of TFCW on immunohistochemistry assessment in the aorta

**Fig. 2:**
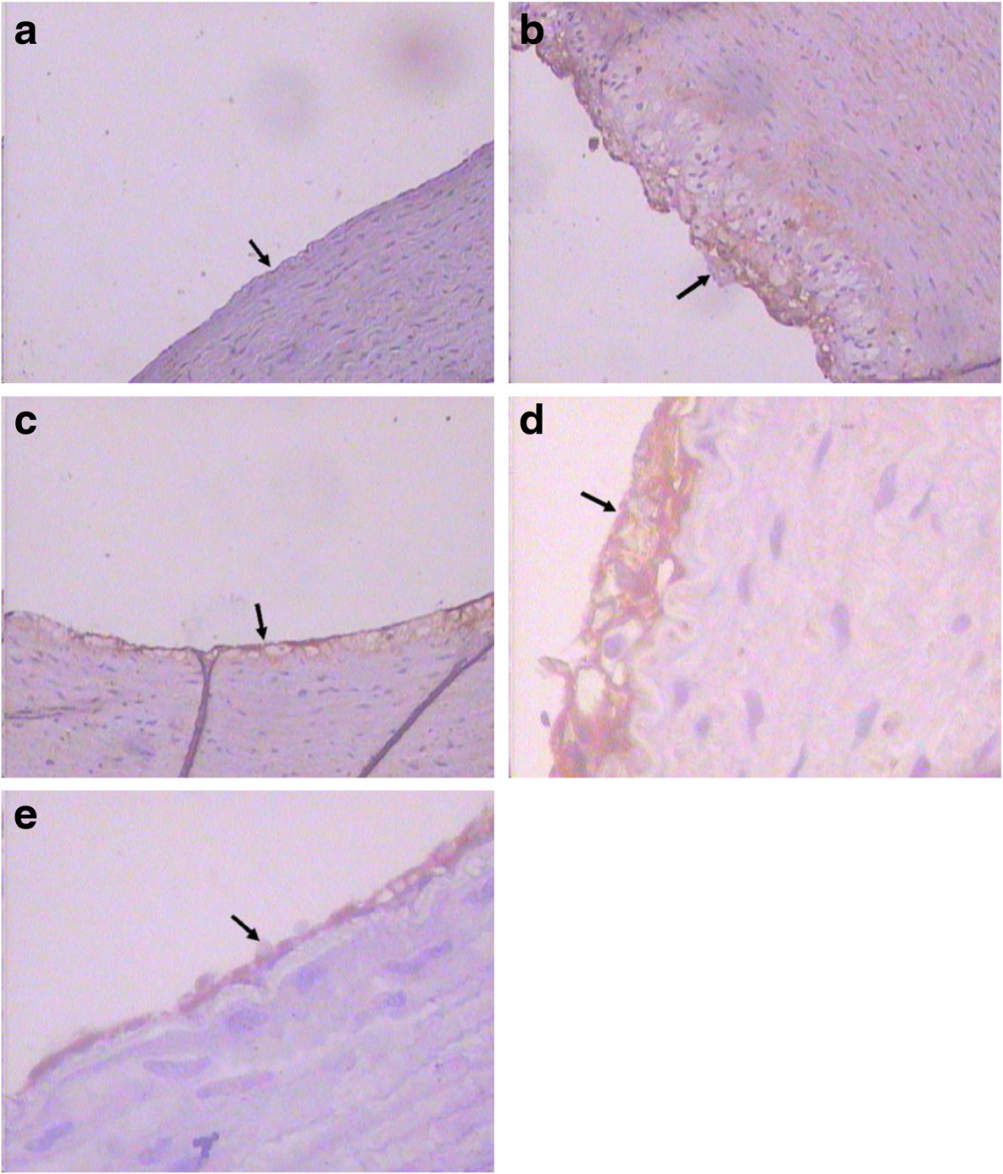
The effect of TFCW on VCAM-1 levels of aorta in atherosclerotic rabbits. **a** Normal control; (**b**) atherosclerotic group; (**c**) positive control (simvastatin 20 mg/kg); (**d**) low dose TFCW (25 mg/kg); (**e**) high dose TFCW (50 mg/kg). (Magnification 400×)

Immunohistochemical evaluation of VCAM-1 expression in aorta section and histomorphometric analysis from experimental groups were shown in Fig. 2. Atherogenic group showed strong VCAM-1 staining, compared to normal control group. Simvastatin and low and high dose TFCW groups showed less VCAM-1 expression than did atherogenic group, which indicated that TFCW may have a preventive effect in atherogenic rabbits.

### Identification of LAB

Seven gram-positive, catalase-negative bacilli were isolated from TFCW. Most of the bacilli had yellow or white colonies with uneven edges and a rough and dull surface. Some of the colonies in the center were translucent, with neat edges. The type colony size was approximately 0.5-3.0 mm. Morphology of the separated *Lactobacillus* species was rod-shaped, slender short or long, and the majority of *Lactobacillus* exhibited chain-like arrangement. We successfully isolated and purified seven isolates of LBA and identified them as *L. brevis, L. kefianofaciens, L. helveticus, L. casei, L. plantarum, L. kefiri* and *Lactococcus lactic* based on morphological characteristics, physiological tests, biochemical tests, and 16S rDNA and 16S rRNA sequence homology with NCBI Reference Sequences NC 008497.1, NC 015602.1, NC 006814.3, NC 008526.1, NC 004567.2, NC 015428.1 and NC 017486. All the test results and sequence homology were shown in Table 3, while all of the sequences were as follows:

**Table 3:** Biochemical characteristics of LAB isolated from TFCW

Tests	1	2	3	4	5	6	7
Catalase test	−	−	−	−	−	−	−
Oxidase test	−	−	−	−	−	−	−
Indole test	−	−	−	−	−	−	−
Trehalose	−	+	+	+	+	+	+
Amygdalin	−	−	−	+	+	+	+
Common temperature (in air)	+	+	+	+	+	+	−
45 °C (in air)	−	−	−	−	+	+	−
15 °C (in air)	−	−	−	+	+	+	+
Growth in 6.5 % NaCl	−	−	−	−	−	−	−
Grouth in pH 9.6	−	−	−	−	−	−	−
Grouth in pH 4.5	+	+	+	+	+	+	+
Aerogenesis by sodium gluconate fermentation	+	N	−	+	N	−	−
Sorbitol	−	−	−	+	+	−	−
Glucose	+	+	+	+	+	+	+
Mannose	−	−	+	+	+	+	+
Arabinose	+	−	−	−	−	−	−
Esculine	−	+	−	+	+	+	+
16S rDNA,16S rRNA sequence homology level (%)	99	99	100	99	99	99	99
Melezitose	−	−	−	+	+	+	−
Fructose	+	−	+	+	+	+	+
Salicin	−	−	−	+	+	+	+
Sodium gluconate	+	−	−	+	+	−	−
Ribose	+	−	+	+	+	+	+
GelatinDeliquescence test	−	−	−	−	−	−	−
Xylose	+	−	−	−	−	−	+
Rhamnose	−	−	−	−	−	−	−
Maltose	+	+	−	+	+	+	+
Lactose	−	+	+	+	+	+	+
Raffinose	−	+	−	−	+	−	−
Aerogenesisbyglucose fermentation	+	−	N	N	N	N	N
Melibiose	+	−	−	−	+	−	−
Galactose	+	+	+	+	+	+	+
Mannitol	−	+	−	+	+	−	+
Sucrose	−	+	−	+	+	+	+
Cellobiose	−	−	−	+	+	+	+

Note: + positive;−negative; Not performed

1. *L. brevis:*
  GCTGACTCCCGAAGGTTATCTCACCGGCTTTGGGTGTTACAAACTCTCATGGTGTGACGGGCGGTGTGTACAAGGCCCGGGAACGTATTCACCGCGGCATGCTGATCCGCGATTACTAGCGATTCCAACTTCATGTAGGCGAGTTGCAGCCTACAATCCGAACTGAGAACGGCTTTAAGAGATTAGCTTAGCCTCACGACTTCGCGACTCGTTGTACCGTCCATTGTAGCACGTGTGTAGCCCAGGTCATAAGGGGCATGATGATTTGACGTCATCCCCACCTTCCTCCGGTTTGTCACCGGCAGTCTCACCAGAGTGCCCAACTGAATGCTGGCAACTGATAATAAGGGTTGCGCTCGTTGCGGGACTTAACCCAACATCTCACGACACGAGCTGACGACAACCATGCACCACCTGTCATTCTGTCCCCGAAGGGAACGTCTTATCTCTAAGATTGGCAGAAGATGTCAAGACCTGGTAAGGTTCTTCGCGTAGCTTCGAATTAAACCACATGCTCCACCGCTTGTGCGGGCCCCCGTCAATTCCTTTGAGTTTCAACCTTGCGGTCGTACTCCCCAGGCGGAGTGCTTAATGCGTTAGCTGCAGCACTGAAGGGCGGAAACCCTCCAACACTTAGCACTCATCGTTTACGGCATGGACTACCAGGGTATCTAATCCTGTTCGCTACCCATGCTTTCGAGCCTCAGCGTCAGTTACAGACTAGACAGCCGCCTTCGCCACTGGTGTTCTTCCATATATCTACGCATTCCACCGCTACACATGGAGTTCCACTGTCCTCTTCTGCACTCAAGTCTCCCAGTTTCCGATGCACTTCTCCGGTTAAGCCGAAGGCTTTCACATCAGACTTAAAAAACCGCCTGCGCTCGCTTTACGCCCAATAAATCCGGACAACGCTTGCCACCTACGTATTACCGCGGCTGCTGGCACGTAGTTAGCCGTGGCTTTCTGGTTAAATACCGTCAACCCTTGAACAGTTACTCTCAAAGGTGTTCTTCTTTAACAACAGAGTTTTACGAGCCGAAACCCTTCTTCACTCACGCGGCATTGCTCCATCAGACTTTCGTCCATTGTGGAAGATTCCCTACTGCTGCCTCCCGTAGGAGTTTGGGCCGTGTCTCAGTCCCAATGTGGCCGATTACCCTCTCAGGTCGGCTACGTATCATCGTCTTGGTGGGCCTTTACCTCACCAACTAACTAATACGCCGCGGGATCATCCAGAAGTGATAGCCGAANCCACCTTTCAAACAAAATCCATGCGGATTTTGTTGTTATACGGTATTAGCACCTGTTTCCAAGTGTTATCCCCTGCTTCTGGGCAGATTTCCCACGTGTTACTCACCAGTTCGCCACTCGCTTCATTGTTGAAATCAANGCAAGCACGTCATTCAACGGAAGCTCGTTCGACT
2. *L. kefianofaciens:*
  CTGCTTAGACGGCTCCTTCCTTGCGGTTAGGCCACCGGCTTTGGGCATTGCAGACTCCCATGGTGTGACGGGCGGTGTGTACAAGGCCCGGGAACGTATTCACCGCGGCGTGCTGATCCGCGATTAATAGCTATTCCAGCTTCGTGCAGTCGAGTTGCAGACTGCAGTCCGAACTGAGAACAGCTTTCAGAGAATTGCTTGCCTTTGCAGGCTCGCTGCTCGTTGTGCTGCCCATTGTAGCACGTGTGTAGCCCAGGTCATAAGGGGCATGATGACTTGACGTCATCCCCACCTTCCTCCGGTTTGTCACCGGCAGTCTCATTAGAGTGCCCAACTTAATGCTGGCAACTAATAACAAGGGTTGCGCTCGTTGCGGGACTTAACCCAACATCTCACGACACGAGCTGACGACAGCCATGCACCACCTGTCTTAGCGTCCCCGAAGGGAACTTTGTATCTCTACAAATGGCACTAGATGTCAAGACCTGGTAAGGTTCTTCGCGTTGCTTCGAATTAAACCACATGCTCCACCGCTTGTGCGGGCCCCCGTCAATTCCTTTGAGTTTCAACCTTGCGGTCGTACTCCCCAGGCGGAGTGCTTAATGCGTTAGCTGCAGCACTGAGAGGCGGAAGCCTCCCAACACTTAGCACTCATCGTTTACGGCATGGACTACCAGGGTATCTAATCCTGTTCGCTACCCATGCTTTCGAGCCTCAGCGTCAGTTGCAGACCAGAGAGCCGCCTTCGCCACTGGTATTCTTCCATATATCTACGCATTCCACCGCTACACATGGAGTTCTACTCTCCTCTTCTGCACTCAAGAAAAACAGTTTCCGATGCAATTCCTCGGTTAAGCCGAGGGCTTTCACATCAGACTTATTCTTCCGCCTGCGCTCGCTTTACGCCCAATAAATCCGGACAACGCTTGCCACCTACGTATTACCGCGGCTGCTGGCACGTAGTTAGCCGTGACTTTCTGGTTGATTACCGTCAAATAAAGGCCAGTTACTACCTCTATCCTTCTTCACCAACAACAGAGCTTTACGGTCCGAAAACCTTCTTCACTCACGCGGCGTTGCTCCATCAGACTTGCGTCCATTGTGGAAGATTCCCTACTGCTGCCTCCCGTANGAGTTTGGGCCGTGTCTCAGTCCCAATGTGGCCGATCAGTCTCTCAACTCGGCTATGCATCACTGCCTTGGTAGGCCGTTACCTTACCAACTAGCTAATGCACCGCGGGTCCATCCTTTAGCGACAGCTTGCGCCGCCTTTTAAAAGCTGTTCATGCGAACTGCTTTCTTATCCGGTATTAGCACCTGTTTCCAAGTGGTATCCCAGACTTAAGGGCAGGTTCCCCACGTGTTACTCACCCATCCGCCGCTCGCTTTCCCAGCGTCCTCACCGAAGTGATTCTGCTGGTTCCGCTCGCTCGACTTGCATGTATTAG
3. *L. helveticus:*
  TTAGACGGCTCCTTCCCGAAGGTTAGGCCACCGGCTTTGGGCATTGCAGACTTCCATGGTGTGACGGGCGGTGTGTACAAGGCCCGGGAACGTATTCACCGCGGCGTTCTGATCCGCGATTACTAGCGATTCCAGCTTCGTGCAGTCGAGTTGCAGACTGCAGTCCGAACTGAGAACAGCTTTCAGAGATTCGCTTGCCTTCGCAGGCTCGCTTCTCGTTGTACTGTCCATTGTAGCACGTGTGTAGCCCAGGTCATAAGGGGCATGATGACTTGACGTCATCCCCACCTTCCTCCGGTTTGTCACCGGCAGTCTCATTAGAGTGCCCAACTTAATGCTGGCAACTAATAACAAGGGTTGCGCTCGTTGCGGGACTTAACCCAACATCTCACGACACGAGCTGACGACAGCCATGCACCACCTGTCTTAGCGTCCCCGAAGGGAACTCCTAATCTCTTAGGATGGCACTAGATGTCAAGACCTGGTAAGGTTCTTCGCGTTGCTTCGAATTAAACCACATGCTCCACCGCTTGTGCGGGCCCCCGTCAATTCCTTTGAGTTTCAACCTTGCGGTCGTACTCCCCAGGCGGAGTGCTTAATGCGTTAGCTGCAGCACTGAGAGGCGGAAACCTCCCAACACTTAGCACTCATCGTTTACGGCATGGACTACCAGGGTATCTAATCCTGTTCGCTACCCATGCTTTCGAGCCTCAGCGTCAGTTGCAGACCAGAGAGTCGCCTTCGCCACTGGTGTTCTTCCATATATCTACGCATTCCACCGCTACACATGGAGTTCCACTCTCCTCTTCTGCACTCAAGAAAAACAGTTTCCGATGCAGTTCCTCGGTTAAGCCGAGGGCTTTCACATCAGACTTATTCTTCCGCCTGCGCTCGCTTTACGCCCAATAAATCCGGACAACGCTTGCCACCTACGTATTACCGCGGCTGCTGGCACGTAGTTAGCCGTGACTTTCTGGTTGATTACCGTCAAATAAAGGCCAGTTACTACCTCTATCCTTCTTCACCAACAACAGAGCTTTACGATCCGAAAACCTTCTTCACTCACGCGGCGTTGCTCCATCAGACTTGCGTCCATTGTGGAAGATTCCCTACTGCTGCCTCCCGTAGGAGTTTGGGCCGTGTCTCAGTCCCAATGTGGCCGTTCAGTCTCTCAACTCGGCTATGCATCATTGCCTTGGTAAGCCGTTACCTTACCAACTAGCTAATGCACCGCGGGGCCATCCCATAGCGACAGCTTACGCCGCCTTTTATAAGCTGATCATGCGATCTGCTTTCTTATCCGGTATTAGCACCTGTTTCCAAGTGGTATCCCAGACTATGGGGCAGGTTCCCCACGTGTTACTCACCCATCCGCCGCTCGCGTCCCCAGCATCATTACCGAAGTAAATCTGCTGGTTCTGCTCGCTCGACTGCATGTAT
4. *L. casei:*
  TAGACGGCTCGCTCCCTAAAAGGGTTACGCCACCGGCTTCGGGTGTTACAAACTCTCATGGTGTGACGGGCGGTGTGTACAAGGCCCGGGAACGTATTCACCGCGGCGTGCTGATCCGCGATTACTAGCGATTCCGACTTCGTGTAGGCGAGTTGCAGCCTACAGTCCGAACTGAGAATGGCTTTAAGAGATTAGCTTGACCTCGCGGTCTCGCAACTCGTTGTACCATCCATTGTAGCACGTGTGTAGCCCAGGTCATAAGGGGCATGATGATTTGACGTCATCCCCACCTTCCTCCGGTTTGTCACCGGCAGTCTTACTAGAGTGCCCAACTAAATGCTGGCAACTAGTCATAAGGGTTGCGCTCGTTGCGGGACTTAACCCAACATCTCACGACACGAGCTGACGACAACCATGCACCACCTGTCATTTTGCCCCCGAAGGGGAAACCTGATCTCTCAGGTGATCAAAAGATGTCAAGACCTGGTAAGGTTCTTCGCGTTGCTTCGAATTAAACCACATGCTCCACCGCTTGTGCGGGCCCCCGTCAATTCCTTTGAGTTTCAACCTTGCGGTCGTACTCCCCAGGCGGAATGCTTAATGCGTTAGCTGCGGCACTGAAGGGCGGAAACCCTCCAACACCTAGCATTCATCGTTTACGGCATGGACTACCAGGGTATCTAATCCTGTTCGCTACCCATGCTTTCGAGCCTCAGCGTCAGTTACAGACCAGACAGCCGCCTTCGCCACTGGTGTTCTTCCATATATCTACGCATTTCACCGCTACACATGGAGTTCCACTGTCCTCTTCTGCACTCAAGTTTCCCAGTTTCCGATGCGCTTCCTCGGTTAAGCCGAGGGCTTTCACATCAGACTTAAAAAACCGCCTGCGCTCGCTTTACGCCCAATAAATCCGGATAACGCTTGCCACCTACGTATTACCGCGGCTGCTGGCACGTAGTTAGCCGTGGCTTTCTGGTTGGATACCGTCACGCCGACAACAGTTACTCTGCCGACCATTCTTCTCCAACAACAGAGTTTTACGACCCGAAAGCCTTCTTCACTCACGCGGCGTTGCTCCATCAGACTTGCGTCCATTGTGGAAGATTCCCTACTGCTGCCTCCCGTANNAGTTTGGGCCGTGTCTCAGTCCCAATGTGGCCGATCAACCTCTCAGTTCGGCTACGTATCATCGCCTTGGTGAGCCATTACCTCACCAACTAGCTAATACGCCGCGGGTCCATCCAAAAGCGATAGCTTACGCCATCTTTCAGCCAAGAACCATGCGGTTCTTGGATCTATGCGGTATTAGCATCTGTTTCCAAATGTTATCCCCCACTTAAGGGCAGGTTACCCACGTGTTACTCACCCGTCCGCCACTCGTTCCATGTTGAATCTCGGTGCAAGCACCGATCATCAACGAGAACTCGTTCGACTGCATGTATAGC
5. *L. plantarum:*
  GGCGTGCCTAATACATGCAAGTCGAACGAACTCTGGTATTGATTGGTGCTTGCATCATGATTTACATTTGAGTGAGTGGCGAACTGGTGAGTAACACGTGGGAAACCTGCCCAGAAGCGGGGGATAACACCTGGAAACAGATGCTAATACCGCATAACAACTTGGACCGCATGGTCCGAGTTTGAAAGATGGCTTCGGCTATCACTTTTGGATGGTCCCGCGGCGTATTAGCTAGATGGTGGGGTAACGGCTCACCATGGCAATGATACGTAGCCGACCTGAGAGGGTAATCGGCCACATTGGGACTGAGACACGGCCCAAACTCCTACGGGAGGCAGCAGTAGGGAATCTTCCACAATGGACGAAAGTCTGATGGAGCAACGCCGCGTGAGTGAAGAAGGGTTTCGGCTCGTAAAACTCTGTTGTTAAAGAAGAACATATCTGAGAGTAACTGTTCAGGTATTGACGGTATTTAACCAGAAAGCCACGGCTAACTACGTGCCAGCAGCCGCGGTAATACGTAGGTGGCAAGCGTTGTCCGGATTTATTGGGCGTAAAGCGAGCGCAGGCGGTTTTTTAAGTCTGATGTGAAAGCCTTCGGCTCAACCGAAGAAGTGCATCGGAAACTGGGAAACTTGAGTGCAGAAGAGGACAGTGGAACTCCATGTGTAGCGGTGAAATGCGTAGATATATGGAAGAACACCAGTGGCGAAGGCGGCTGTCTGGTCTGTAACTGACGCTGAGGCTCGAAAGTATGGGTAGCAAACAGGATTAGATACCCTGGTAGTCCATACCGTAAACGATGAATGCTAAGTGTTGGAGGGTTTCCGCCCTTCAGTGCTGCAGCTAACGCATTAAGCATTCCGCCTGGGGAGTACGGCCGCAAGGCTGAAACTCAAAGGAATTGACGGGGGCCCGCACAAGCGGTGGAGCATGTGGTTTAATTCGAAGCTACGCGAAGAACCTTACCAGGTCTTGACATACTATGCAAATCTAAGAGATTAGACGTTCCCTTCGGGGACATGGATACAGGTGGTGCATGGTTGTCGTCAGCTGGTGTCGTGAGATGTTGGGTTAAGTCCCGCAACGAGCGCAACCCTTATTATCAGTTGCCACCATTAAGTTGGGCACTCTGGTGAGACTGCCGGTGACAAACCGGAGGAAGGTGGGGATGACGTCAAATCATCATGCCCCTTATGACCTGGGCTACACACGTGCTACAATGGATGGTACAAGGAGTTGCGAACTCGCGAGAGTAAGCTAATCTCTTAAAGCCATTCTCAGTTCGGATTGTAGGCTGCAACTCGCCTACATGAAGTCGGAATCGCTAGTAATCGCGGATCAGCATGCCGCGGTGAATACGTTCCCGGGCCTTGTACACACCGCCCGTCACACCATGAGAGTTTGTAACACCCAAAGTCGGTGGGGTAACCTTTTAGGAACCAGCCGCCTAAGGTGG
6. *L. kefiri:*
  CTTAGACGGCTGGTCCCCGAAGGTTACCTCACCGGCTTTGGGTGTTACAAACTCTCATGGTGTGACGGGCGGTGTGTACAAGGCCCGGGAACGTATTCACCGTGGCATGCTGATCCACGATTACTAGCGATTCCAACTTCATGCAGGCGAGTTGCAGCCTGCAATCCGAACTGAGAACGGCTTTAAGAGATTAGCTTGACCTCGCGGTTTCGCGACTCGTTGTACCGTCCATTGTAGCACGTGTGTAGCCCAGGTCATAAGGGGCATGATGATTTGACGTCATCCCCACCTTCCTCCGGTTTGTCACCGGCAGTCTTGCTAGAGTGCCCAACTGAATGCTGGCAACTAACAATAAGGGTTGCGCTCGTTGCGGGACTTAACCCAACATCTCACGACACGAGCTGACGACAACCATGCACCACCTGTCATTCTGTCCCCGAAGGGAACGCCTAATCTCTTAGGTTGGCAGAAGATGTCAAGACCTGGTAAGGTTCTTCGCGTAGCATCGAATTAAACCACATGCTCCACCGCTTGTGCGGGCCCCCGTCAATTCCTTTGAGTTTCAACCTTGCGGTCGTACTCCCCAGGCGGAGTGCTTAATGCGTTAGCTGCAGCACTGAAGGGCGGAAACCCTCCAACACTTAGCACTCATCGTTTACGGCATGGACTACCAGGGTATCTAATCCTGTTCGCTACCCATGCTTTCGAGCCTCAGCGTCAGTTACAGACCAGACAGCCGCCTTCGCCACTGGTGTTCTTCCATATATCTACGCATTTCACCGCTACACATGGAGTTCCACTGTCCTCTTCTGCACTCAAGTCTCCTGGTTTCCGATGCACTTCTCCGGTTAAGCCGAAGGCTTTCACATCAGACCTAAGAAACCGCCTGCGCTCGCTTTACGCCCAATAAATCCGGACAACGCTTGCCACCTACGTATTACCGCGGCTGCTGGCACGTAGTTAGCCGTGGCTTTCTGGTTGGATACCGTCAAGATGTCAACAGTTACTCTGACACCTGTTCTTCTCCAACAACAGAGTTTTACGAGCCGAAACCCTTCATCACTCACGCGGCGTTGCTCCATCAGACTTTCGTCCATTGTGGAAGATTCCCTACTGCTGCCTCCCGTAGGAGTTTGGGCCGTGTCTCAGTCCCAATGTGGCCGATTACCCTCTCAGGTCGGCTACGTATCATTGCCTTGGTAGGCCATTACCTTACCAACAAGCTAATACGCCGCGGGTCCATCCTAAAGTGATAGCCGAAGCCATCTTTTAAACCAAAACCATGTGGTTTTGGTTGTTATACGGTATTAGCACCTGTTTCCAAGTGTTATCCCCTACTTCAAGGGCAGGTTACCCACGTGTTACTCACCAGTTCGCCACTCGTTTCGTGTTAAATCATTTAAATGCAAGCATCTAAAATCAATAACGGAAACGCGTTCGACTTGCATGTAT
7. *Lactococcus lactic:*
  GGTCTTACCTTAGGAAGCGCCCTCCTTGCGGTTAGGCAACCTACTTCGGGTACTCCCAACTCCCGTGGTGTGACGGGCGGTGTGTACAAGGCCCGGGAACGTATTCACCGCGGCGTGCTGATCCGCGATTACTAGCGATTCCGACTTCATGTAGGCGAGTTGCAGCCTACAATCCGAACTGAGAATGGTTTTAAGAGATTAGCTAAACATCACTGTCTCGCGACTCGTTGTACCATCCATTGTAGCACGTGTGTAGCCCAGGTCATAAGGGGCATGATGATTTGACGTCATCCCCACCTTCCTCCGGTTTATCACCGGCAGTCTCGTTAGAGTGCCCAACTTAATGATGGCAACTAACAATAGGGGTTGCGCTCGTTGCGGGACTTAACCCAACATCTCACGACACGAGCTGACGACAACCATGCACCACCTGTATCCCGTGTCCCGAAGGAACTTCCTATCTCTAGGAATAGCACGAGTATGTCAAGACCTGGTAAGGTTCTTCGCGTTGCTTCGAATTAAACCACATGCTCCACCGCTTGTGCGGGCCCCCGTCAATTCCTTTGAGTTTCAACCTTGCGGTCGTACTCCCCAGGCGGAGTGCTTATTGCGTTAGCTGCGATACAGAGAACTTATAGCTCCCTACATCTAGCACTCATCGTTTACGGCGTGGACTACCAGGGTATCTAATCCTGTTTGCTCCCCACGCTTTCGAGCCTCAGTGTCAGTTACAGGCCAGAGAGCCGCTTTCGCCACCGGTGTTCCTCCATATATCTACGCATTTCACCGCTACACATGGAATTCCACTCTCCTCTCCTGCACTCAAGTCTACCAGTTTCCAATGCATACAATGGTTGAGCCACTGCCTTTTACACCAGACTTAATAAACCACCTGCGCTCGCTTTACGCCCAATAAATCCGGACAACGCTCGGGACCTACGTATTACCGCGGCTGCTGGCACGTAGTTAGCCGTCCCTTTCTGGGTAGTTACCGTCACTTGATGAGCTTTCCACTCTCACCAACGTTCTTCTCTACCAACAGAGTTTTACGATCCGAAAACCTTCTTCACTCACGCGGCGTTGCTCGGTCAGACTTTCGTCCATTGCCGAAGATTCCCTACTGCTGCCTCCCGTANGAGTTTGGGCCGTGTCTCAGTCCCAATGTGGCCGATCACCCTCTCAGGTCGGCTATGTATCATCGCCTTGGTGAGCCTTTACCTCACCAACTAGCTAATACAACGCGGGATCATCTTTGAGTGATGCAATTGCATCTTTCAAACTTAAAACTTATGTTTAAAGTTGTTATGCGGTATTAGCATTCGTTTCCAAATGTTGTCCCCCGCTCAAAGGCAGATTCCCCACGCGTTACTCACCCGTTCGCTGCTCTTCAAATTGGTGCAAGCACCAATCTTCATCGCTCAACTTGCATGATTAG

### Identification of probiotic yeasts

We identified two yeast isolates to be *S. unisporus* and *I. orientalis* based on morphological characteristics, physiological and biochemical tests and 26S rDNAD1/D2 sequence homology with sequences AY 707865 and EU 019220 in Genbank. All the test results and sequence homology were shown in Table 4, while all of the sequences were as follows:

**Table 4:** Biochemical characteristics of yeasts isolated from TFCW

Test	A	B
Glucose	+	+
Galactose	−	−
Sucrose	−	−
Maltose	−	−
Lactose	−	−
Raffinose	−	−
Arabinose	−	−
Cellobiose	−	−
Esculin	−	−
Fructose	−	−
Mannitol	−	−
Mannose	−	−
Melibiose	−	−
Rhamnose	−	−
Salicin	−	−
Sorbose	−	−
Xylose	−	−
MushroomCarbohydrates	−	−
D-Ribose	−	−
Carbon Assimilation tests	−	−
Galactose	+	−
Sucrose	−	−
Maltose	−	−
Cellobiose	−	−
Trehalose	−	−
Lactose	−	−
Raffinose	−	−
Soluble starch	−	−
D-xylose	−	−
L-arabinose	−	−
L-arabinose	−	−
D-ribose	−	−
L-Rhamnose	−	−
Erythrosealcohol	−	−
Adonitol	−	−
D-mannitol	−	−
Succinate	−	+
Citric acid	−	+
Inositol	−	−
Nitrogen Assimilation test		−
Nitrate	−	−
Hydrochloric cadaverine	+	+
L-Lysine	+	−
Growth in medium vitamin free	−	+
Growth at 37 °C	+	+
Colony size (μm)	(2.5−4.5) × (3.6−6.0)	(2.4−3.6 × (3.6−6.0)
26SrDNAD1/2sequencehomology level (%)	99	99

Note: + positive;−negative. A and B are different species of yeast

1. *S. unisporus:*
  TGCATATTCAATAAGCGGAGGAAAAGAAACCAACCGGGATTGCCTTAGTAACGGCGAGTGAAGCGGCAAAAGCTCAAATTTGAAATCTAGTACCTTCGGTGCTCGAGTTGTAATTTGTAGAGGGATACTTTGGGGCCGTTCCTTGTCTATGTTCCTTGGAACAGGACGTCATAGAGGGTGAGAATCCCGTGTGGCGAGGAGTGCGGTTCTATGTAAAGTGCCTTCGAAGAGTCGAGTTGTTTGGGAATGCAGCTCTAAGTGGGTGGTAAATTCCATCTAAAGCTAAATATTGGCGAGAGACCGATAGCGAACAAGTACAGTGATGGAAAGATGAAAAGAACTTTGAAAAGAGAGTGAAAAAGTACGTGAAATTGTTGAAAGGGAAGGGCATTTGATCAGACATGGTGTTTTGCGCCCTCTGCTCCTTGTGGGTGGGGGAATCTCGCAGCTCACTGGGCCAACATCAGTTTTGGTGGTCGGATAAATCCGTAGGAATGTGGCTTGCCTCGGCAAGTGTTATAGCCTGCGGGAATACGGCCAGCTGGGACTGAGGACTGCCACTTTTGTCAAGGATGTTGGCATAATGGTTATATGCCGCCCGTCTTGAAACACGGACCAA
2. *I. orientalis:*
  GCATATCAATAAGCGGAGGAAAAGAAACCAACAGGGATTGCCTCAGTAGCGGCGAGTGAAGCGGCAAGAGCTCANATTTGAAATCGTGCTTTGCGGCACGAGTTGTAGATTGCAGGTTGGAGTCTGTGTGGAAGGCGGTGTCCAAGTCCCTTGGAACAGGGCGCCCAGGAGGGTGAGAGCCCCGTGGGATGCCGGCGGAAGCAGTGAGGCCCTTCTGACGAGTCGAGTTGTTTGGGAATGCAGCTCCAAGCGGGTGGTAAATTCCATCTAAGGCTAAATACTGGCGAGAGACCGATAGCGAACAAGTACTGTGAAGGAAAGATGAAAAGCACTTTGAAAAGAGAGTGAAACAGCACGTGAAATTGTTGAAAGGGAAGGGTATTGCGCCCGACATGGGGATTGCGCACCGCTGCCTCTCGTGGGCGGCGCTCTGGGCTTTCCCTGGGCCAGCATCGGTTCTTGCTGCAGGAGAAGGGGTTCTGGAACGTGGCTCTTCGGAGTGTTATAGCCAGGGCCAGATGCTGCGTGCGGGGACCGAGGACTGCGGCCGTGTAGGTCACGGATGCTGGCAGAACGGCGCAACACCGCCCGTCTTGAAACACGGACCA

## Discussion

Major finding of the current study is that treatment with TFCW significantly modified lipid profile and reduced CRP, ICAM-1 and VCAM-1 in atherosclerotic rabbit model. Preventive effects of TFCW in atherogenic rabbits were also demonstrated by reduction in VCAM-1 expression and formation of atheromatous plaques on aortic endothelium. In fact, accumulation of cholesterol and lipids leads to foam cell formation, which is regarded as a critical process in development of atherosclerosis [12]. Overwhelmingly strong evidence demonstrated that integrated dysregulation of serum lipidic and inflammatory components in vascular wall contributes to an early and advanced atherosclerotic development [13]. VCAM-1 is a critical mediator of adhesion and uptake of monocytes across the endothelium in the early stages of atherosclerosis development [14], which mediates the assembly of monocytes, macrophages, T lymphocytes and platelets and their adherence to vascular wall that plays a key role in pathogenesis of atherosclerosis [15]. CRP, a phylogenetically highly conserved plasma protein, is the classical acute phase reactant in humans, and preliminary evidence for interaction of CRP with lipids implicates a possible relationship between CRP and atherosclerosis [16].

In this study, 7 potential probiotic lactobacillus species, including *L. casei* [17], *L. helveticus* [18], *L. plantarum* [19] and *L. lactis* [20], which are proven probiotics, were identified in the TFCW. These LAB species may be responsible for the protective effect of TFCW against atherosclerosis in atherogenic rabbits. Indeed, LAB increase immune response [21] and reduce cholesterol [22, 23] both in animal models [24, 25] and humans [26]. LAB or LAB with active bile salt hydrolase have been suggested to lower cholesterol through interaction with host bile salt metabolism [27].

In addition, goat milk fermented with *Lactocillus fermenterum* ME-3 improves antioxidant activities in human blood, thus providing antiatherogenic activity [28]. Consumption of probiotic-containing dairy food reduces cholesterol possibly through degradation of cholesterol, and probiotic lactobacilli and their metabolic by-products lower cholesterol and provide preventive and therapeutic effects against ischemic heart syndromes [6, 29].

We also identified two probiotic yeasts in TFCW. *S. unisporus* is ubiquitously present in fermented milk, cheese and kefir-based milk products and may produce vitamins and interact with LAB, which may enhance LAB growth [30]. *S. unisporus* contains middle chain fatty acids up to C 14:0 to 18:1 and produces a high percentage of palmitoleate. Palmitoleic acid, an omega-7 monounsaturated fatty acid, is a major constituent of human adipose tissues and is considered antioxidant [31]. *I.orientalis* exhibits a higher tolerance for pH, bile, and heat stress for survival in gastrointestinal environment as a probiotic [32]. *I. orientalis* commonly exists in cheeses and other fermentation milk products and exhibits ability to scavenge 1,1 diphenyl-2-picrylhydrazyl and to inhibit lipid peroxidation, thus presenting antioxidant activity as a potential probiotic in fermented milk products [33].

Notably, oxidized LDL in vascular wall seems to be a key factor in atherosclerosis, because oxidized LDLs might recruit monocytes and favor their transformation into foam cells through a receptor-mediated intake (scavenger pathway). Moreover, cytotoxic oxidized form of LDLs are likely responsible for endothelial cell damage and macrophage degeneration in atherosclerotic human plaque [34]. Polyunsaturated fat decreases TC and LDL-C by lowering LDL-C production rates and/or increasing LDL clearance rates [35, 36]. Consequently, omega-3polyunsaturated fatty acid (ω3-PUFA) has beneficial effects in preventing atherosclerotic diseases, and a strong positive correlation prevails between intake of saturated fatty acids and an increased incidence of CVD [37]. Therefore, inhibition of oxidation of unsaturated fatty acids is of significance for the prevention of atherosclerosis and/or CVD [38]. Furthermore, hypercholesterolemia is a major risk factor for the development of atherosclerosis [39]. Thus, we speculate that TFCW exerts its anti-atherogenic effect possibly through the identified probiotic LAB and yeasts. However, atherosclerotic effect of each individual LAB was not investigated in this study, and further studies of such effects by each kind of LAB and the possible underlying mechanisms are necessary.

## Conclusions

In conclusion, current study indicates that 7 LAB and 2 yeasts identified from TFCW and TFCW have significant anti-atherosclerotic potential in atherosclerotic rabbits and may modulate lipid metabolism and protect aorta in the atherosclerotic condition, which might be related to various probiotics acting through reducing the CRP, VCAM-1 and ICAM-1 levels and protecting the aortic endothelium.

## Abbreviations

BW: Body weight
CRP: C-reactive protein
CVD: Cardiovascular disease
HDL-C: High density lipoprotein cholesterol
HPF: High power field
HTST: High temperature short time
ICAM-1: Intercellular adhesion molecule-1
LAB: Lactic acid bacteria
LDL-C: Low-density lipoprotein cholesterol
MRS: Man Rogosa Sharpe
PPAR-γ: Peroxisome proliferator-activated receptor-γ
SPF: Specific pathogen free
TC: Total cholesterol
TFCW: Traditional fermented cheese whey
TG: Triglycerides
VCAM-1: Vascular cell adhesion molecule-1
